# Hyalase in Dermatology: Applications Beyond Filler Management

**DOI:** 10.1111/jocd.70543

**Published:** 2025-11-09

**Authors:** Saba Hasanzadeh, Parisa Farokh, Golsa Sadat Hosseini, Maryam Abbasian, Ifa Etesami, Shirin Zaresharifi, Sahar Dadkhahfar

**Affiliations:** ^1^ Skin Research Center Shahid Beheshti University of Medical Sciences Tehran Iran; ^2^ Department of Science Rutgers, the State University of New Jersey New Brunswick New Jersey USA; ^3^ Autoimmune Bullous Diseases Research Center Razi Hospital, Tehran University of Medical Sciences Tehran Iran; ^4^ Department of Dermatology Loghman Hakim Hospital, Shahid Beheshti University of Medical Sciences Tehran Iran; ^5^ Department of Dermatology Shohada‐e Tajrish Hospital, Shahid Beheshti University of Medical Sciences Tehran Iran

**Keywords:** dermatology, extravasation, fibrosis, GVHD, hyaluronidase, keloid, microstomia

## Abstract

**Background:**

Hyaluronic acid (HA) is a key extracellular matrix component in the skin. Hyaluronidase is an enzyme that breaks down HA into monosaccharides. The FDA has approved this enzyme for hypodermoclysis, drug absorption enhancement, and subcutaneous urography. The most common application of this enzyme is the dissolution of HA fillers.

**Aim:**

This study aimed to explore the various applications of hyaluronidase beyond its common medical uses. Moreover, we illustrate the mechanism by which this enzyme is helpful in conditions such as drug extravasation, scleroderma, scleromyxedema, and keloids.

**Methods:**

A comprehensive review of hyaluronidase applications in medicine, with a specific focus on dermatology, was conducted using PubMed/MEDLINE, the Cochrane Library, and Embase. The conditions under which hyaluronidase was applied are discussed, and the protocols used for each condition are evaluated and summarized.

**Results:**

Hyaluronidase improves tissue permeability and enhances drug delivery. Therefore, it is beneficial for treating keloids, managing pain, and enhancing the distribution of chemotherapeutic agents within tumors. It is also useful for reducing fibrosis in scleroderma, scleromyxedema, and chronic GVHD. Hyaluronidase is considered a safe drug, and its adverse effects are infrequent.

**Conclusion:**

Hyaluronidase has the potential to be useful for various medical and dermatological conditions. Further research is required to fill existing gaps and establish standardized usage protocols.

## 1 Introduction

Hyaluronic acid (HA), a hydrophilic glycosaminoglycan, is a key component of the normal interstitial barrier and the extracellular matrix (ECM). It contributes to the organization of collagen, fibrin, and fibronectin during cell migration. HA also plays a role in inflammation, angiogenesis, and wound healing [1, 2, 3, 4].

Human hyaluronidase is an endoglycosidase found in various body fluids and organs, including the skin, eyes, liver, spleen, kidneys, placenta, uterus, and testis. It decomposes hyaluronic acid into monosaccharides by splitting its glycosidic bonds [5].

Hyaluronidase is classified in many ways. There are four main categories, including the classification based on the source of the enzyme (eukaryotes, prokaryotes, and viruses), amino acid sequence homology, optimal reaction pH (acidic and neutral), and specificity and catalytic mode. Meyer et al. classified hyaluronidases into three types based on their specificity and catalytic mechanisms, including EC 3.2.1.35 (hyaluronoglucosaminidase), which is mainly derived from mammals and venoms. It hydrolyzes β−1,4‐glycosidic bonds in HA. EC 3.2.1.36 (hyaluronoglucosidase) is derived from leech saliva. This type targets the β−1,3‐glycosidic bond in HA. EC 4.2.2.1 (hyaluronate lyase) is produced by microbial organisms and breaks β−1,4‐glycosidic bonds in HA. Therefore, various types of hyaluronidases follow distinct degradation pathways, resulting in different end products [6].

Several brands of hyaluronidase are currently available on the market, including some FDA‐approved drugs such as Hylenex (Halozyme Therapeutics Inc., San Diego, CA, USA), which is a recombinant human hyaluronidase produced using genetically engineered Chinese hamster ovary cells, with a concentration of 150 U per milliliter [7].

Amphadase (Amphastar Pharmaceuticals Inc., Rancho Cucamonga, CA, USA) is a bovine‐derived hyaluronidase extracted from bovine testes, also containing 150 U per milliliter [8].

Vitrase (ISTA Pharmaceuticals, Irvine, CA, USA) is an ovine hyaluronidase obtained from ovine testes, with each milliliter containing 200 units [9]. Hydase (PrimaPharm Inc., San Diego, CA, USA) is another bovine testicular hyaluronidase formulation, providing 150 units per milliliter [10]. Each product has a different potency and dosage that must be considered during administration.

Hyaluronidase has been used in medical applications since 1949 [11]. The US Food and Drug Administration has authorized the following indications for this enzyme: hypodermoclysis, enhancement of drug absorption and diffusion in the skin, and facilitation of the resorption of radiopaque agents in subcutaneous urography. Furthermore, the European Union has licensed hyaluronidase for the reabsorption of subcutaneous hematomas [5].

Hyaluronidase is utilized for various off‐label purposes in different medical fields. It is commonly employed in dermatology to dissolve hyaluronic acid‐based dermal fillers [11], but it has many other applications. This article aims to review the different applications of hyaluronidase, providing clinicians with a comprehensive understanding of the potential roles of this enzyme in various medical contexts.

## 2 Materials and Methods

We conducted a literature search for information on the applications of hyaluronidase in dermatology from papers published until November 2024. The search was performed using the PubMed/MEDLINE, Cochrane Library, and Embase databases with the keywords (hyaluronidase), (dermatology), (skin), and ((hyaluronidase) AND ((skin) OR (dermatology))). This review specifically focuses on the applications of hyaluronidase in dermatology, excluding studies on its applications in other fields of medicine. Due to the limited number of articles in this area, which mainly consist of case reports and lack clinical trials, we undertook a review and summary of the available case reports.

### 2.1 Extravasation

Extravasation injury occurs when fluid from an intravenous (IV) line escapes into adjacent tissue spaces. The incidence of extravasation is estimated to be between 0.1% and 6.5% in hospitalized patients. Children are at increased risk of extravasation because they have small, fragile veins, lower peripheral circulation, and flexible subcutaneous tissue [12]. Extravasation is diagnosed based on local pain, swelling, edema, burning sensation, and lack of venous return. Extravasation injuries cause skin ulceration and necrosis, followed by necrosis of deeper tissues [13, 14, 15]. The extent of necrosis is influenced by the characteristics of the extravasated agent, which encompasses the type, concentration, and volume of the injected substance [16]. Medications with high osmolarity, low pH, and high molecular weight cause more severe extravasation injury [17]. Factors such as age, level of consciousness, competency of the venous system, and the type and location of the IV cannula determine the risk of extravasation injury [18]. Treatment of this condition depends on the agent and its volume and degree of tissue damage [19]. If this condition is overlooked, surgical resection and debridement may be necessary [15].

By breaking down hyaluronic acid and subcutaneous tissue bonds, hyaluronidase reduces the hyaluronic acid viscosity, increases tissue permeability, and facilitates the dispersion and absorption of the extravasated substance [15]. This process helps to minimize tissue damage caused by toxic drugs by creating larger areas in the subcutaneous tissue [5]. Optimal results are achieved when hyaluronidase is administered subcutaneously or intradermally within 1 h after an extravasation injury. Nevertheless, hyaluronidase treatment can still be effective up to 24 h after such an injury [20]. Extravasation can cause cosmetic and functional complications [21]. Therefore, prompt treatment is crucial to avoid adverse effects.

Several studies reported the administration of hyaluronidase in patients with extravasation injuries [22, 23, 24, 25, 26, 27, 28, 29, 30, 31, 32, 33, 34, 35, 36, 37, 38]. A study evaluated ten neonates with peripheral intravenous extravasation injuries using point‐of‐care ultrasound in the neonatal intensive care unit and described the alterations in tissue before and after treatment with hyaluronidase. The authors measured the extent of fluid dispersion and the reduction in edema. Ultrasound revealed bright blood collection in the subcutaneous tissue in one infant. Following the administration of hyaluronidase, the collection in the tissue became less echogenic, and the pressure‐related edema was reduced, spreading over a wider area. The findings aligned with physical examination, which confirms the response to therapy [23].

In another report, hyaluronidase was used for the management of compartment syndrome in a female patient with high‐grade glioma who underwent craniotomy and received 100 mL of 20% mannitol intravenously over 30 min. This resulted in edema and a large vesicle in her limb below the elbow, leading to a diagnosis of compartment syndrome secondary to mannitol extravasation. Subcutaneous injection of hyaluronidase was administered in several regions surrounding the catheter site. The limb edema progressively diminished, and both the radial pulse and blisters improved over time. In this instance, hyaluronidase effectively addressed mannitol‐induced compartment syndrome, eliminating the need for fasciotomy [26].

Studies that used hyaluronidase for the treatment of extravasation are summarized in Table 1.

**TABLE 1:** The management and outcome of using hyaluronidase in extravasation.

Study	Design	Group of study	Extravasated drug	Management	Outcome
Boyar et al., 2018 [23]	Case series	Neonates (*n* = 10)	D10W TPN PRBC	Hyaluronidase	Improvement of edema and skin elevation
Davies et al., 1994 [24]	Case report	Infants (*n* = 2)	Parental nutrition	Subcutaneous hyaluronidase (500–1000 U) and saline flushes	Rapid healing with minimal scar
Fox et al., 2017 [25]	Case report	60‐year‐old man	Amiodarone	Intradermal hyaluronidase (0.1 mL of the 150 U/mL solution)	Decreased erythema, warmth, swelling and pain
Sokol et al., 1998 [33]	Case report	14‐month‐old boy	Phenytoin	Subcutaneous hyaluronidase (0.2 mL fractions of 15 U/mL solution in four sites, then repeated)	Decrease in swelling and redness. Pulses could be auscultated
Martin et al., 1994 [30]	Case report	4‐month‐old girl	Corrosive drugs used during cardiac resuscitation	Hyaluronidase (1500 U), liposuction, and saline washout	No signs of soft tissue damage after 2 weeks
Kaur et al., 2021 [26]	Case report	50‐year‐old female	Mannitol	Subcutaneous hyaluronidase (150 U in 10 mL normal saline), 1 mL in each site	Reduction in swelling, Improvement of radial pulse volume
Kumar et al., 2003 [28]	Case report	51‐year‐old man	50 mL of 20% mannitol	150 U of hyaluronidase, mixed in 10 mL of normal saline	The swelling subsided completely by the next day
Napoli et al., 2005 [31]	Case series	Adult patients (*n* = 25)	Anticancer drugs	Infiltration of saline solution/Intradermic or subcutaneous local anesthetic and hyaluronidase, aspiration with lipoaspiration cannulas	Very small hyperpigmented areas/one temporary edema No infections, hematomas, excessive bleeding, skin paraesthesias, scar retractions, or other significant complications were seen
Yan et al., 2014 [32]	Retrospective study	Neonates (*n* = 13)	TPN, CaCl_2_, dextrose 10% water Intravenous immunoglobulin PAMBA + etamsylate	150 U/mL of hyaluronidase and topical hirudoid	Treatment prevented further damage to the tissue, reduced swelling and erythema
Khan et al., 2002 [27]	Retrospective study	Adult patients (*n* = 18)	Cytotoxic agents^a^ Phenytoin Flucloxacillin	Kit containing (30‐g 1% hydrocortisone cream, one hyaluronidase injection of 1500 U intravenously, one 5‐mL 1% lignocaine injection, one 250‐mL 0.9% sodium chloride infusion)	No plastic surgical intervention required
Rens et al., 2021 [35]	Case report	Male infant	Dextrose 10% water	Hyaluronidase diluted with 1 mL of sodium chloride 0.9%	After 2 weeks, wound healed successfully without any functional effects
Wiegand et al., 2010 [36]	Case report	17‐year‐old girl	Dextrose 50% water	Hyaluronidase (150 U/1 mL)	Improvement of symptoms, no complications
Lawson et al., 2013 [29]	Case report	46‐year‐old man	100 mL of 50% dextrose (D50% W)	Subcutaneous hyaluronidase	Decrease in swelling, redness and pain
Rowlett et al., 2012 [32]	Case report	57‐year‐old woman	Iodinated contrast (iohexol)	Five 150 U vials of recombinant human hyaluronidase in 150 U aliquots	Full recovery without any sequelae
Merendonk et al., 2021 [34]	Case series	Adult patients (*n* = 2)	Remdesivir	Hyaluronidase was diluted with 0.9% sodium chloride injection to a concentration of 150 U/mL	Mark improvement in signs and symptoms
Zenk et al., 1981 [38]	Case series	Infants (*n* = 2)	Nafcillin	Subcutaneous hyaluronidase, 15 U in 1 mL of normal saline	The area healed completely
Bertelli et al., 1994 [22]	Case series	Patients (*n* = 7)	Vinca alkaloids	Hyaluronidase	No skin necrosis after treatment with hyaluronidase

Abbreviations: PAMBA, para‐aminomethylbenzoic acid; PRBC, packed red blood cell; TPN, total parental nutrition.

^a^ Plaxitel, methotrexate, DTIC, MVP, docetaxel, doxorubicin, epirubicin, cisplatin, taxotere.

### 2.2 Scleroderma

Scleroderma is a chronic autoimmune disease that affects the connective tissue. Microvascular fibroproliferative vasculopathy and immune dysregulation are the main pathogenesis of the disease. However, the etiology of this condition remains unknown. In this condition, the ECM components accumulate excessively, particularly hyaluronan (HA), collagen types I, III, VII, and fibronectin in the skin and internal organs [39, 40]. As a result, fibrosis occurs in the skin, which is the main feature of scleroderma [41]. The skin fibrosis around the mouth results in microstomia, a common cutaneous manifestation of scleroderma, which makes chewing, speaking, and maintaining oral hygiene difficult [39]. There are some drugs available that block the pro‐fibrosis pathways, including D‐penicillamine, IFN‐γ, Relaxin, Bovine CI, Halofuginone, Pirfenidone, Minocycline, and Inhibitors of Rho kinases. However, there is still not sufficient medication for fibrosis elimination [41]. Nevertheless, hyaluronidase has some off‐label uses for improving sclerosis in sclerodermic patients.

Abbas et al. reported a case of a 60‐year‐old woman with 18 years of morphea, including jaw involvement for 3 years, leading to a 110‐pound weight loss due to eating difficulties. She received 150 units of intradermal hyaluronidase with 1 mL of bacteriostatic saline at the most affected site, repeated over four sessions, every 2 weeks. Post‐treatment, her vertical oral opening improved from 2 to 5 cm, with no adverse effects [42].

In a separate investigation, Kumar et al. reported the case of a 45‐year‐old female patient with microstomia as a result of systemic scleroderma. The condition was managed through a regimen involving the administration of 1500 U of hyaluronidase and 4 mg of dexamethasone sodium phosphate, injected weekly into the affected areas for 5 months. Following this treatment, the patient's mouth opening increased from 2.5 to 3.8 cm [43].

A case report described the treatment of a 53‐year‐old woman with microstomia due to limited cutaneous systemic sclerosis, who received 470 U of hyaluronidase injections over 7 months. Her Mouth Handicap in Systemic Sclerosis score improved from 42 to 38, leading to greater comfort during eating and dental procedures [39].

Additional details are provided in Table 2.

**TABLE 2:** The management, outcome, and adverse effects of using hyaluronidase in microstomia.

Study	Design	Group of study	Management	Outcome	Adverse effects
Abbas et al., 2019 [42]	Case report	60‐year‐old woman	Four sessions, intradermal injections of hyaluronidase (0.05–0.1 mL) were administered at 5 mm intervals in areas where indurations were detected. A 150 U vial of hyaluronidase was reconstituted with 1 mL of saline for each session	Vertical opening of mouth: Before treatment = 20 mm After treatment = 50 mm	None
Kumar et al., 2016 [43]	Case report	45‐year‐old woman	Injections of hyaluronidase mixed with corticosteroids were administered every 2 weeks for 2 months, followed by weekly injections for an additional 2 months. injection consisted of 1500 U of hyaluronidase combined with 4 mg of dexamethasone sodium phosphate	Mouth opening: Before treatment = 25 mm After treatment = 38 mm	None
Melvin et al., 2019 [39]	Case report	53‐year‐old woman	The safety and efficacy were evaluated during the first 2 months with intralesional injections of 20 U and 50 U hyaluronidase undiluted, respectively. After observing no adverse reactions and confirming efficacy, the patient received diluted in saline total of 400 U hyaluronidase, administered over the subsequent 2 months	Mouth Handicap in Systemic Sclerosis (MHISS) score: Before treatment = 42 of 48 After treatment = 38 of 48	Mild bruising on the left side

### 2.3 Scleromyxedema

Scleromyxedema, also known as diffuse or generalized sclerodermoid lichen myxedematosus, is a primary cutaneous mucinosis characterized by a widespread papular and sclerodermoid skin eruption, typically associated with monoclonal gammopathy [44]. The histopathological features of this disease include fibroblast proliferation, fibrosis, and excessive mucin deposition. It is assumed that the dysregulated cytokine stimulation of glycosaminoglycan synthesis and fibroblast proliferation or plasma cell‐associated monoclonal gammopathy may play a role in the manifestations of the disease [45].

Some systemic approaches to treating the disease include intravenous immunoglobulin, thalidomide, corticosteroids, and some other drugs. In addition, there have been some reports of using lasers such as CO_2_ and YAG, dermabrasion, and electrocoagulation for localized treatment of skin lesions for cosmetic purposes [45].

Microstomia is a manifestation of scleromyxedema in patients with facial involvement, as shown in a case reported by Akaslan et al. In this case, a 69‐year‐old man developed microstomia despite receiving various medications. Following a single session of perioral injection of 1500 U of hyaluronidase, the patient experienced an improvement in mouth opening [46].

### 2.4 GVHD

Graft‐versus‐host disease (GVHD) is an immune‐mediated reaction that occurs in patients with hematological malignancies who have undergone allogeneic hematopoietic stem cell transplantation [47]. Skin manifestations of GVHD include a wide range of sclerotic and nonsclerotic presentations [48]. Given the diverse and varying severity of GVHD manifestations, different medications are administered for treatment, including corticosteroids and immunotherapeutic agents [49]. Hyaluronidase is another agent used to treat sclerotic lesions resulting from GVHD. Cheng et al. reported a case of a 35‐year‐old man with melanoma and Hodgkin lymphoma who developed chronic GVHD during the treatment period, presenting with morphea‐like plaques on his arms and chest. He received hyaluronidase injections without dilution in 5 sessions at 1 to 4‐week intervals, targeting the firmest areas of the plaque on his right arm at 1 cm intervals. The initial dose was 480 U, which was increased to 960 U in subsequent sessions, with a total dose of 4080 U administered. The fibrotic area of the arm showed increased flexibility post‐injections, with initial improvements noted 14 days after the first injection, and the patient did not experience adverse reactions [50].

### 2.5 Keloid

Keloids are raised scars that can develop around any skin injury after healing. They result from the excessive deposition of type I and III collagen fibers [51]. Keloids develop gradually over several months and can be significantly larger than the initial wound [52, 53].

Triamcinolone acetonide is the first‐line treatment for keloids. However, several other treatments are available, including botulinum toxin, 5‐fluorouracil, verapamil, and bleomycin. Scientists are investigating treatments that can be used individually or in combination to enhance the outcomes of keloids [54]. Hyaluronidase is a promising agent used as an adjunct to other treatments to improve effectiveness.

Hyaluronidase decreases collagen deposition in the ECM by degrading HA. Moreover, it has been suggested that hyaluronidase alters the fibroblast secretory process. This leads to reduced collagen synthesis. This mechanism may contribute to the therapeutic effects observed in conditions such as keloids [5, 55].

In a study involving 20 patients with keloids or hypertrophic scars, participants received injections of a solution containing 0.6 mL of 5‐fluorouracil (250 mg/5 mL), 0.4 mL of triamcinolone acetonide (40 mg/mL), and 1500 IU of hyaluronidase. The mixture was injected into the body of the scar, with an average of three injection sessions conducted at one‐month intervals. This treatment decreased the symptoms and flattened the keloids. This inexpensive and readily available treatment had no systemic side effects [56].

In a randomized trial, 100 patients were divided into five treatment groups to compare the efficacy of triamcinolone acetonide alone versus its combination with hyaluronidase or radiofrequency, intralesional verapamil hydrochloride, and intralesional radiofrequency. Among the 20 patients treated with an injection of triamcinolone acetonide (40 mg/mL) and hyaluronidase (1500 IU/mL) in a 1:1 ratio, sixteen patients completed the study, which included up to eight sessions at three‐week intervals. Of these, 11 patients (68.75%) achieved complete lesion removal. In this study, intralesional triamcinolone acetonide, its combination with radiofrequency, and hyaluronidase were found to be almost equally effective. However, triamcinolone acetonide combined with hyaluronidase is considered safer due to its lower side effects [57].

In another randomized trial, Parmar et al. divided 160 patients into three groups to receive intralesional triamcinolone acetonide alone or in combination with hyaluronidase or radiofrequency. The results indicated that all three treatment modalities were nearly equally effective. However, the combination of intralesional triamcinolone acetonide and hyaluronidase (triamcinolone 40 mg/mL with hyaluronidase 1500 IU/mL in a 1:1 ratio) showed the fewest side effects, with only 18.75% of patients experiencing atrophy. In contrast, atrophy was observed in 31.25% of patients receiving triamcinolone alone [58].

Keloids can be effectively treated using a variety of methods, such as laser therapy, drug monotherapy, and combination therapy. Each treatment option offers unique benefits, allowing patients to access the most appropriate care for their conditions [59].

In combination therapy, the use of hyaluronidase has shown effective results with fewer side effects, making it the preferred choice.

## 3 Discussion

Hyaluronidase is an enzyme with remarkable versatility and therapeutic potential. It depolymerizes hyaluronic acid, the main component of the ECM, leading to changes in the interstitial matrix. It has broad off‐label applications in medicine, dermatology, and aesthetics [5, 60]. Hyaluronidase breaks down hyaluronic acid in the ECM and increases tissue permeability. As a result, it enhances drug delivery to the tissue. Therefore, it is used as a spreading agent to enhance drug diffusion [61].

When hyaluronidase is given alongside anticancer agents, it improves the penetration of the drug into malignant tumors [62]. Hyaluronidase contributes to alleviating pain in patients suffering from chronic issues, such as radiculopathy and low back pain [63]. In a randomized controlled trial, patients receiving hyaluronidase with local anesthetic showed a significantly lower postoperative pain score than those receiving only local anesthetic during carpal tunnel release [64]. Moreover, hyaluronidase enhances peribulbar, retrobulbar, and sub‐Tenon's anesthesia during cataract surgery. It enables rapid onset of anesthesia and pain relief with reduced anesthetic quantities, minimizing side effects [65]. It has been demonstrated that the level of hyaluronic acid is increased in transplanted organs. Utilizing hyaluronidase helps resolve edema by degrading hyaluronic acid, a factor in developing inflammatory edema [66].

The most common application of hyaluronidase is in cosmetic procedures for dissolving hyaluronic acid fillers. This allows for corrections and resolves complications, including nodule formation, vascular occlusion, and filler misplacement. The physical type of the hyaluronic acid filler, volume, patient factors, location, and indication of use (emergent or non‐emergent situation) are considered for the individual dose needed. Thus, physicians need to consider various factors to evaluate effectiveness [67].

Currently, experiments are being conducted on the applications of hyaluronidase in various aspects, such as wound healing, gene therapy, and melanoma treatment. Hyaluronidase may play a beneficial role in melanoma treatment in two main ways. First, it can enhance the distribution of chemotherapeutic agents by impacting the ECM [68]. Additionally, hyaluronic acid is linked to tumor progression and is associated with poor prognosis in patients with melanoma. It plays a role in the signaling pathways of bone morphogenetic proteins 4 and 7 through the cell‐surface receptor CD44. This interaction promotes the expression of inhibitors of DNA binding/differentiation (Id) proteins 1 and 3, contributing to tumor progression and negative outcomes. Therefore, hyaluronidase may reduce the expression of Id1 and Id3 proteins, potentially helping to mitigate tumor progression [69]. Moreover, hyaluronidase has been effective in delivering plasmid DNA into subcutaneous tumors, such as melanoma, making it a promising candidate for electrogene therapy [70].

The incidence of adverse reactions to hyaluronidase is low. Localized allergic reactions, such as erythema and pruritus, are associated with hyaluronidase. However, a few anaphylactic reactions have also been reported [71]. Immediate hypersensitivity reactions (immunoglobulin E‐mediated, type I) are the most common allergic reactions. However, delayed hypersensitivity reactions (T‐cell–mediated, type IV) can also occur, presenting with delayed signs of allergic response [5].

Several drug interactions with hyaluronidase need to be considered. Some agents that act as hyaluronidase antagonists include dexamethasone, salicylates, indomethacin, diclofenac, antihistamines, antioxidants, flavonoids, heparin, vitamin C, radiologic contrast media, mast cell stabilizers, and dicumarene. Additionally, there are some other medications that may interact with hyaluronidase, including dopamine, α‐adrenergic agonists, phenytoin, furosemide, and benzodiazepines [3].

In patients with a history of allergies to bee stings or bovine collagen, cross‐reactivity may occur due to hyaluronidase injection. Therefore, it is essential to consider the patient's medical history before administering the injection [5].

No significant adverse reactions were reported in any of the articles we reviewed, indicating the safety of hyaluronidase treatment. Although this agent is generally considered safe, clinicians must take the necessary precautions.

Further research is needed to explore the potential medical applications of hyaluronidase. To date, no large‐scale trials have been conducted on the application of this enzyme. Our existing knowledge relies mostly on observational studies, making it difficult to establish effective management protocols. Further studies may provide a deeper understanding of the therapeutic potential of hyaluronidase.

## Author Contributions

Saba Hasanzadeh: acquisition of data, drafting the manuscript. Parisa Farokh: acquisition of data, drafting the manuscript. Golsa Sadat Hosseini: acquisition of data, drafting the manuscript. Maryam Abbasian: acquisition of data, revising the manuscript. Ifa Etesami: acquisition of data, revising the manuscript. Shirin Zaresharifi: acquisition of data, revising the manuscript. Sahar Dadkhahfar: conception and design, acquisition of data, revising the manuscript.

## Ethics Statement

The authors have nothing to report.

## Conflicts of Interest

The authors declare no conflicts of interest.

## Data Availability

Data sharing is not applicable to this article as no datasets were generated or analyzed during the current study.
